# Supplementary material for: Capture‐C reveals preformed chromatin interactions between HIF‐binding sites and distant promoters
Source: EMBO Rep. 2016 Aug 9;17(10):1410–21. doi: 10.15252/embr.201642198 (PMC5048371; doi:10.15252/embr.201642198)
Supplement: Supplementary file 1 — Appendix [file EMBR-17-1410-s001.pdf]

Capture-C reveals preformed chromatin interactions between HIF-binding sites and distant promoters

Platt JL[1], Salama R[1], Smythies J[1], Choudhry H[2], Davies JOJ[3], Hughes JR[3], Ratcliffe PJ[4*], Mole DR[1*]

Figure S1

A

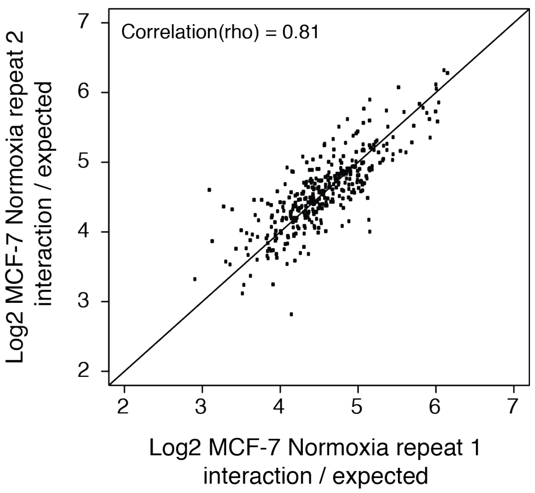

B

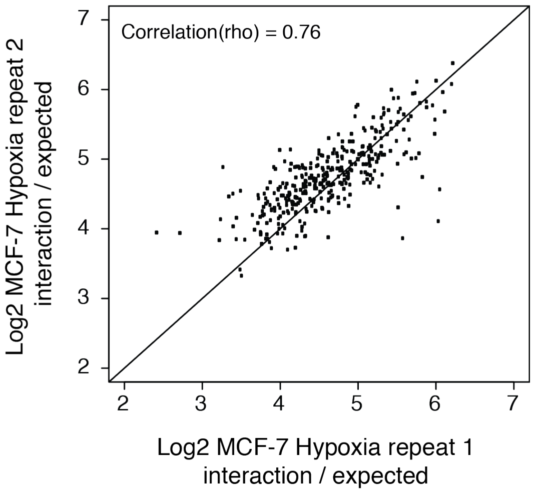

C

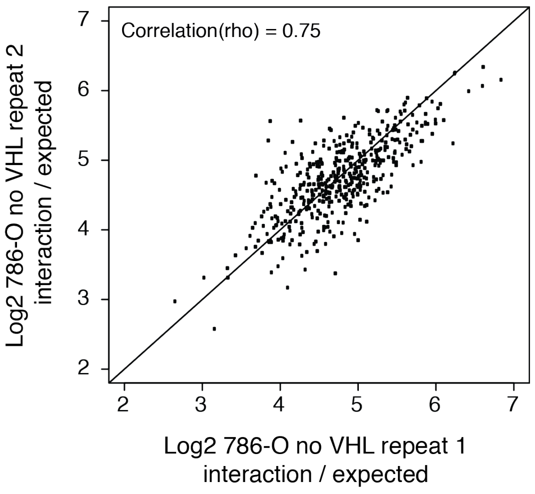

Figure S1: Reproducibility of Capture-C.

Capture-C experiments were performed in duplicate. For each site that looped to a HIF-binding site, the interaction frequencies (normalized to the number of informative reads in each dataset and to the expected distribution of counts at a given distance from the capture site) for the two replicates were plotted against each other for:

- A) MCF-7 cells in normoxia
- B) MCF-7 cells in hypoxia
- C) 786-O cells.

Figure S2

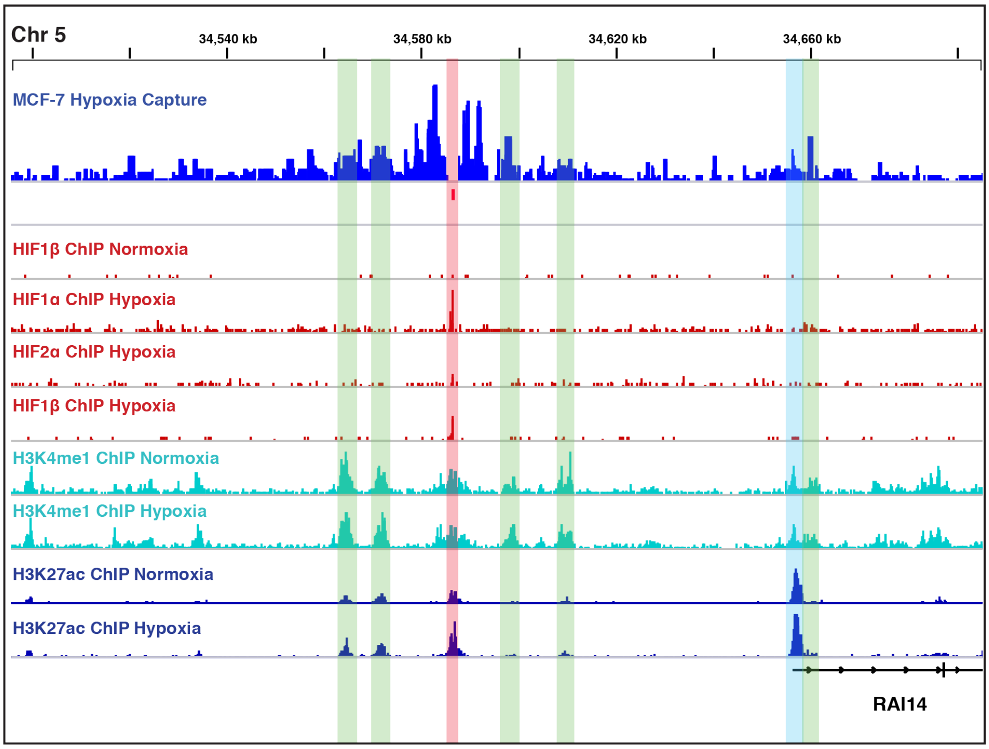

Chr 5
34,540 kb
34,580 kb
34,620 kb
34,660 kb
MCF-7 Hypoxia Capture
HIF1β ChIP Normoxia
HIF1α ChIP Hypoxia
HIF2α ChIP Hypoxia
HIF1β ChIP Hypoxia
H3K4me1 ChIP Normoxia
H3K4me1 ChIP Hypoxia
H3K27ac ChIP Normoxia
H3K27ac ChIP Hypoxia
RAI14

Figure S2: Looping of HIF-binding site to promoters and enhancers.

Capture-C (top) and ChIP-seq tracks for subunits of HIF, H3K4me1, H3K27ac, and RNApol2 at the RAI14 gene locus, in MCF-7 cells. Capture-C was performed using oligonucleotides towards the 'viewpoint' region at the HIF binding site (highlighted in red). Looping to the transcriptional start site of the RAI14 gene is highlighted in blue, looping to other enhancers is highlighted in green.

Figure S3

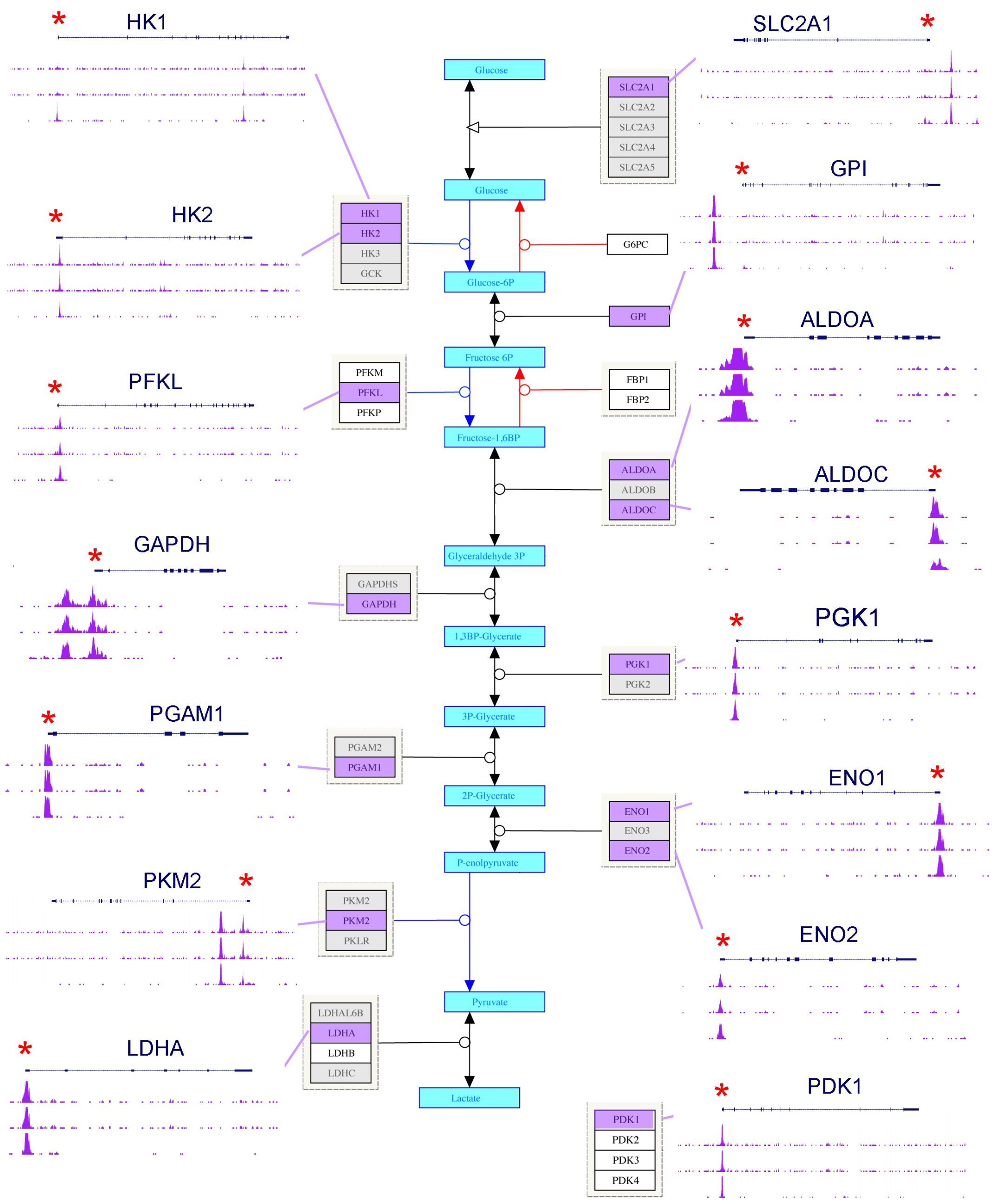

HK1
HK2
PFKL
GAPDH
PGAM1
PKM2
LDHA
SLC2A1
GPI
ALDOA
ALDOC
PGK1
ENO1
ENO2
PDK1
Glucose
Glucose
Glucose-6P
Fructose 6P
Fructose-1,6BP
Glyceraldehyde 3P
1,3BP-Glycerate
3P-Glycerate
2P-Glycerate
P-enolpyruvate
Pyruvate
Lactate
SLC2A1
SLC2A2
SLC2A3
SLC2A4
SLC2A5
HK1
HK2
HK3
GCK
G6PC
GPI
PFKM
PFKL
PFKP
FBP1
FBP2
ALDOA
ALDOB
ALDOC
GAPDHS
GAPDH
PGK1
PGK2
PGAM2
PGAM1
ENO1
ENO3
ENO2
PKM2
PKM2
PKLR
LDHAL6B
LDHA
LDHB
LDHC
PDK1
PDK2
PDK3
PDK4

Figure S3: HIF binding at glycolysis pathway and related genes.

The glycolysis pathway shown with metabolic substrates highlighted in, blue together with the enzymes that catalyze each step. Those enzymes highlighted in grey are not expressed in MCF-7 cells (as judged by lack of signal in RNA-seq analysis). Those highlighted in purple are hypoxia-regulated (in the same RNA-seq analysis). Tracks (in order) for HIF1α, HIF2α and HIF1β ChIP-seq signals are shown in purple for each regulated gene in purple together with the positions of the MCF-7 transcript in blue and the transcriptional start site (red asterix).

Figure S4

A

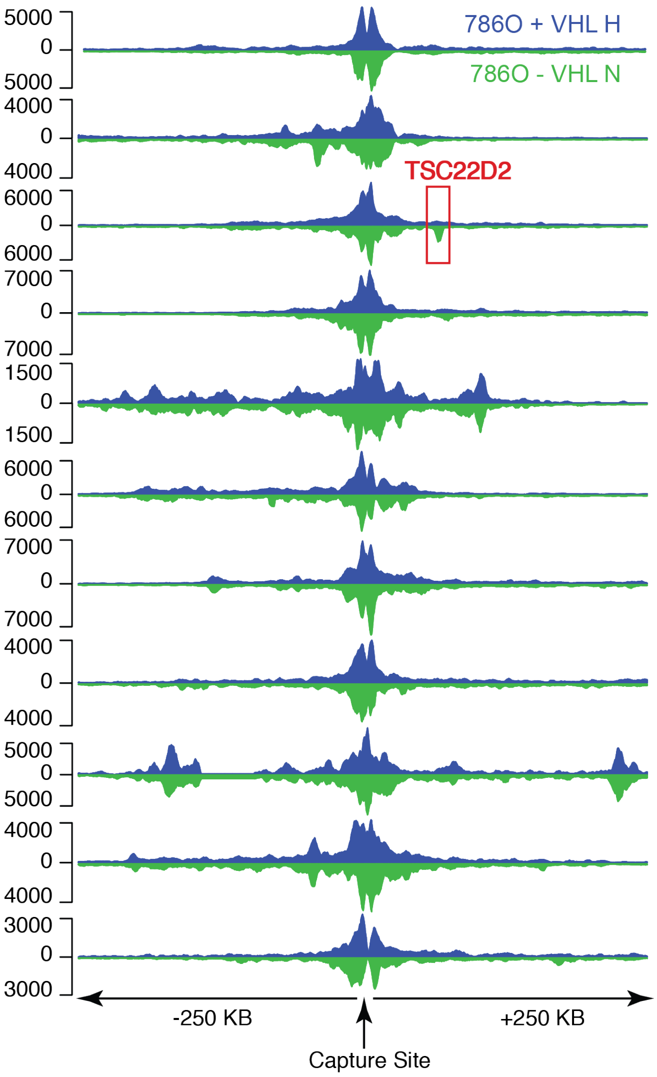
786O + VHL H
786O - VHL N
TSC22D2
5000
0
5000
4000
0
4000
6000
0
6000
7000
0
7000
1500
0
1500
6000
0
6000
7000
0
7000
4000
0
4000
5000
0
5000
4000
0
4000
3000
0
3000
-250 KB
+250 KB
Capture Site

B

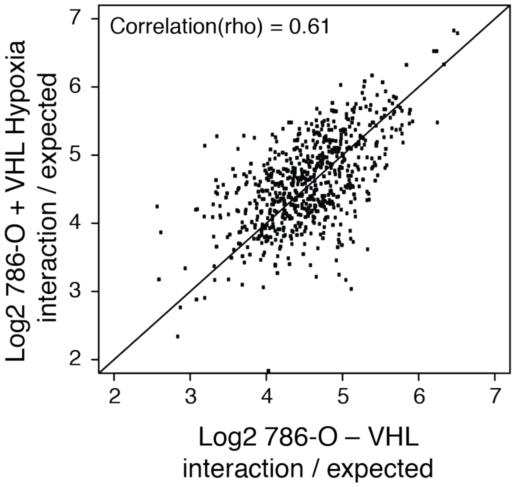
Correlation(rho) = 0.61
Log2 786-O + VHL Hypoxia
interaction / expected
Log2 786-O – VHL
interaction / expected
2
3
4
5
6
7

Figure S4: Chromatin looping is stable after long term HIF stabilisation

- A) Capture-C tracks from hypoxic 786-O cells reconstituted with wtVHL (blue) and 786-O cells with defective VHL (inverted in green) are shown for each HIF-binding site captured. A single promoter site that differentially captured (TSC2202) is highlighted in red.

- B) For each site that looped to a HIF-binding site, the normalized interaction frequency in hypoxic 786-O cells reconstituted with wtVHL (vertical axis) was plotted against that in 786-O cells (horizontal axis).

Figure S5

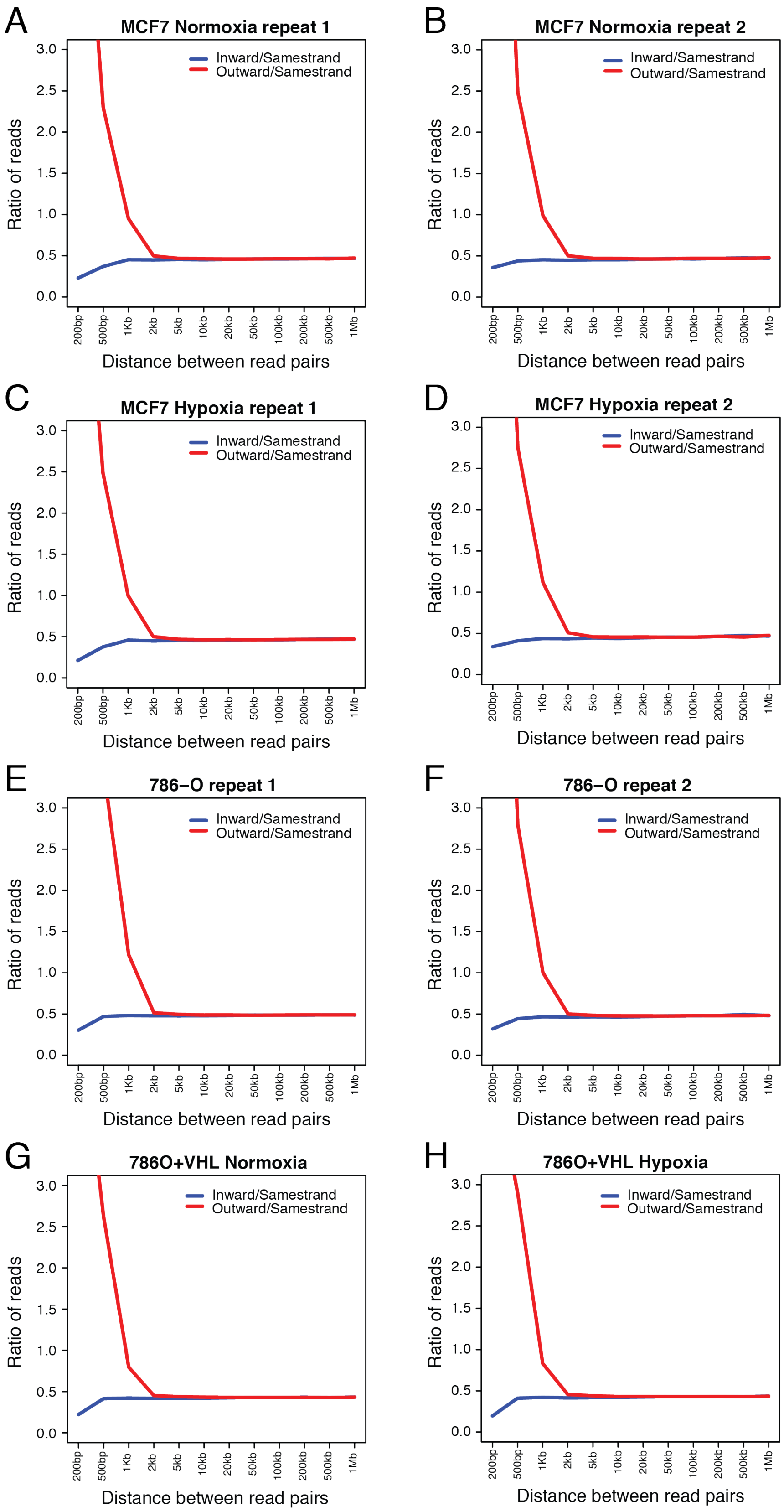
A
MCF7 Normoxia repeat 1
B
MCF7 Normoxia repeat 2
C
MCF7 Hypoxia repeat 1
D
MCF7 Hypoxia repeat 2
E
786–O repeat 1
F
786–O repeat 2
G
786O+VHL Normoxia
H
786O+VHL Hypoxia
Inward/Samestrand
Outward/Samestrand
Ratio of reads
Distance between read pairs
200bp
500bp
1Kb
2kb
5kb
10kb
20kb
50kb
100kb
200kb
500kb
1Mb
0.0
0.5
1.0
1.5
2.0
2.5
3.0

Figure S5: Composition of paired-end Capture-C reads in different experiments as a function of the genomic distance between a pair of reads.

All aligned Dpn2 fragment pairs were identified and the genomic distance between them were calculated as the gap size between the two fragments. For each fragment pairs we identified their relative orientation as being inward, outward or same-strand as previously described[1], and then computed the ratios between the classes of read pairs as indicated relative to genomic distance. Blue line = inward/samestrand; red line = outward/samestrand, plotted against genomic distance between read pairs. The expected ratios are 0.5 if two fragments are truly cut and ligated.

Figure S6

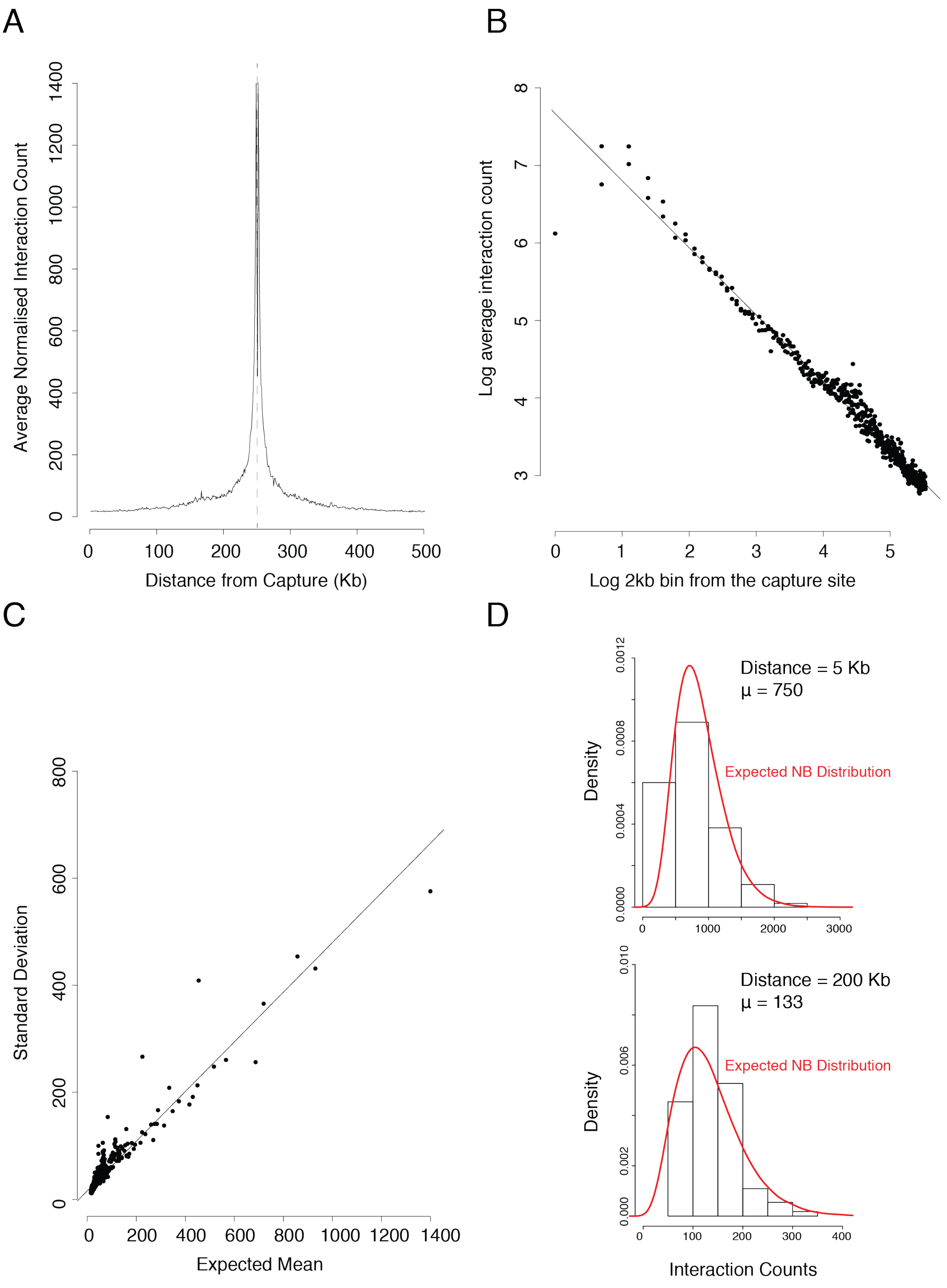
A
Average Normalised Interaction Count
0
200
400
600
800
1000
1200
1400
0
100
200
300
400
500
Distance from Capture (Kb)
B
Log average interaction count
3
4
5
6
7
8
0
1
2
3
4
5
Log 2kb bin from the capture site
C
Standard Deviation
0
200
400
600
800
0
200
400
600
800
1000
1200
1400
Expected Mean
D
Density
0.0000
0.0004
0.0008
0.0012
0
1000
2000
3000
Distance = 5 Kb
μ = 750
Expected NB Distribution
Density
0.000
0.002
0.006
0.010
0
100
200
300
400
Distance = 200 Kb
μ = 133
Expected NB Distribution
Interaction Counts

Figure S6:

- A) Normalized average capture signal (RPKM) across all capture probes ±250 kb. This shows the random expected signal at a specified distance.

- B) Data is aggregated in bins as explained in the methods. The log of the signal in every bin is plotted against the log of the bin's distance to the capture probe.

- C) For every bin, the mean and variance is calculated across all the probes. The index of dispersion (variance/mean) for all the bins is plotted against its mean. This shows a linear relationship, which allowed us to estimate the dispersion parameter of a negative binomial distribution.

- D) The distribution predicted by the model represented in red lines, is overlaid on top of the histogram showing the actual signal at a distance of 5 kb and at a distance of 200 kb. This red line shows a good fit to the actual data.

Figure S7

A

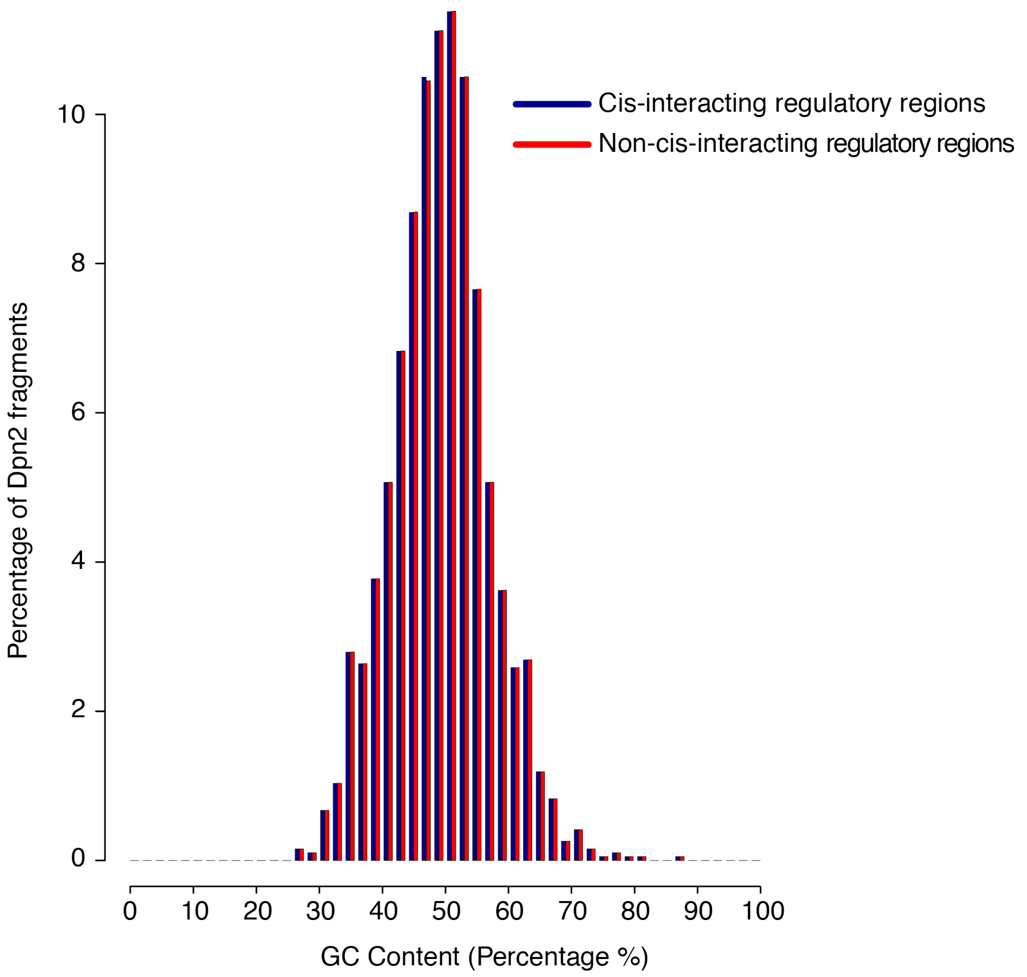

Cis-interacting regulatory regions
Non-cis-interacting regulatory regions
Percentage of Dpn2 fragments
0
2
4
6
8
10
0 10 20 30 40 50 60 70 80 90 100
GC Content (Percentage %)

B

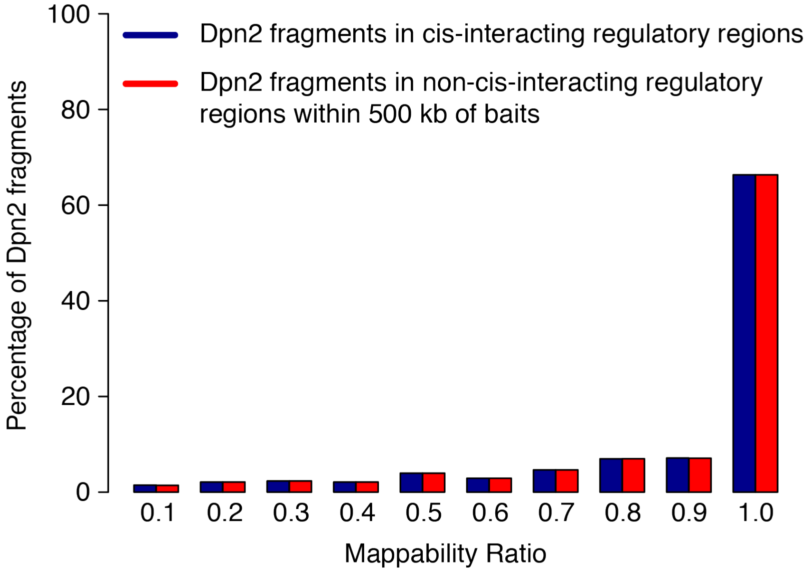

Dpn2 fragments in cis-interacting regulatory regions
Dpn2 fragments in non-cis-interacting regulatory regions within 500 kb of baits
Percentage of Dpn2 fragments
0
20
40
60
80
100
0.1 0.2 0.3 0.4 0.5 0.6 0.7 0.8 0.9 1.0
Mappability Ratio

Figure S7: Effects of GC-content and mappability on the contact frequency

- A) The GC-content of the 200 bases at each end of each Dpn2 fragment was calculated. Frequency distributions were plotted showing the percentage of Dpn2 fragment ends with varying GC-content. In blue are data for statistically-significant cis-interacting regulatory regions. For comparison in red are data for similarly defined regulatory elements within 500 kb of each bait site that did not interact. Cis-interacting and control, non-cis-interacting, regulatory regions had comparable levels of GC-content.

- B) 50-base pseudo-reads were generated every 9 bases within regions up to 200 bases from the end of each Dpn2 fragment similar to previously described[1]. Bowtie was then used to determine the fraction of uniquely mapped pseudo-reads (mappability) expressed on a scale of 0 (no pseudo-reads mapped uniquely) to 1 (all pseudo-reads mapped uniquely). The proportion of Dpn2 fragments with varying degrees of mappability was then plotted as a frequency histogram for Dpn2 fragments within statistically significant cis-interacting regulatory regions (blue bars) and non-cis-interacting regulatory regions within 500 kb of bait sites (red bars). The majority of Dpn2 fragments revealed a high degree of mappability indicating good coverage. Furthermore, comparable levels of mappability were observed between statistically significant cis-interacting regulatory regions and for non-interacting regulatory regions.

Table S1: Bait sites and capture probes

Bait Dpn2 fragment	capture type	Cell type	probe 1 sequence	probe 2 sequence
chr1:115666026-115667357	enhancer	786O	GAT CTA GGA CAT GAG CAT ATA TAA GGC GAT ATG CAT ATG CCC AGG AAA GAC GTG AGA AAG TCC ACA TCT CTC ATC TCT GGC TGA CAT TGA GGC TCT GCA CAG CAA GAA GTG AAG GGT TAG	GAG GCT GTC AAT CAT GAA TTT TAT ATT AAG CAA AAC TAT CCT TCA AAA ATG AAG GAG AGG TTG GGC ACA GTG GCT CAC GCC TGT AAT CCC AGC ACT TTG GGA GGC AGA GGT GGG TGG ATC
chr1:43424102-43424740	promoter	N/A	GAT CCG AAG CCC ATC CCC ACT GCG GCC AGC GGC CCG GCG GGG CGC TGC GCT CGG GAC CCG CAC CGA GCC AGG CTC GGA GAG GCG CGC GGC CCG CCC CGG GCG CAC AGC GCA GCG GGG CGG	GGA CCT CGG GGC GGG GCC TGG GGC CCA GCC TGT CCT GGG CGG CGC TCT GCG GGG AGG GGG TGA GGG GAG TGG CCG GGG CCG GGG CCC CCC CTA ACC ACT GCC TCG CTC AGG CTG CCG ATC
chr1:8086133-8087834	promoter	N/A	GAT CCC GGC GCC CGG CGC GGG GCC CCC TGA GGG AAC GGA GCG CTG CGT GGC CCT CCC CAC CCC CTC AGC GCG CCA GGC CCC TTA CCC CGG AGG AGC GGC GGC TGC CCC GCG GCG CCC TCC	TAG AAC AAA TTC CCT GGC TGG TCC TCT GTT GGG AGA GCC CAG AGA CCT GTC GCT GAC CTC TAG CCT CCT GGA GCC CCT GCT TGC ACT TCA GCA ATA ATG CCT GAT AGT TAG ACA CTG ATC
chr1:8938715-8938952	promoter	N/A	GAT CTC CGT GCT CCG GGT ACC CAC AGA TAC TGT CCG CGC CGC CCG CGC CGA CTT CCT GCC TAG CTA AGG CGA GCC CCA CCC ACT TCC CGC CTC CGG GGC ACG CCC CCT CAG CTC CGC CTC	TCG CCA TTC GTC GGG AGA GCC GTC ACT CAT TCC CTC ACC TCC CGG AAA GGG GTG GGA CTG CGA CGG CTG CAG TAG CGT GGA AAG GGG GGT GAC GTC CCG CGG ACT TGA GGC GGG CAG ATC
chr2:173420581-173420782	promoter	N/A	GAT CTG GGC GGC GGC TGC GGC CTG GCG CGC GCT CCG CAT CTC CTT CCT CGG GAG GCT GGG CGG GAC TCC GGC GAG GGG GCG GGC CCC GCA AGC GCC CGC CCC TTT TCT CTC CCC GCC TCC	GCC CCG CAA GCG CCC GCC CCT TTT CTC TCC CCG CCT CCT TCC CGC TCC GCG TCC CGC CCA GCG GCG CAG GGG GCC GGG CTC CGG CTA GGA GGG TGG GGG CCG CGC CGG TGA CAG CCG ATC
chr2:70823890-70824097	enhancer	786O	GAT CAC ACA TGC TGT TCC CAA CTT CCC TTC CCC TTG ACA CGC TGT GGT GAA CTC TGA ATT CCA GCA CGT CTC CAC TCT GCT GTA GAG CCT TTC TAG GGC TGG CAA GCA TTC TGC CAC AGA	CTT TCT AGG GCT GGC AAG CAT TCT GCC ACA GAG CTG GTC TTT GTG GCT TTA TGC CCA CAG TAA TTA CTA GGG CTG GGC CAT GCA ACC GTC TAA CCT CTT CAA TTT ACA GCC TCC AGG ATC
chr2:75060942-75061218	promoter	N/A	GAT CAC GCT CCC CCC ACC CAT AGC CGA GCC TGA CGC GGC GGT GGC TCA TGC GCC TTT CCG TCC CAG CCT TTA GCC	GTG TCT GAG CGG CCG CGC CCG CGA GCC GTG AGC GAT GAT TGG CTG CGC CAC GGC GGC GGG CGG TCC GTG GGC GCA

			ACG GAC CAC ACG TCC CAT CTC AGG CGC CCC GCC CCT CCC CCG CCC	CAC ACC CTC CCC GCG CAG CCA ATG GGC GTG CGC ACG TCA CTG ATC
chr3:150063120-150063407	enhancer	MCF7 & 786O	GAT CCA GGA TTT CAC CAT ATG CTA TTG GGT CAT ATG TCT CAA AAC TCA GGT GTG CTC ATC GTT GAA CTA ATA ACC TCA GTT ATT CAT TTG GTT TCA AAA TGC TTA TCC TGG CAC GTA CAG	CTG ATA AGC TAT TCT GTG TTT AAA GCA CAT ATT TTT AAA AAA TCA GAG GTC TGC ATC TCC AAG CAC AGG GCA GTC CAG GAT AAA TCA GAC CCA CTA AAA ATA TAA GGA CTA TAG CTG ATC
chr5:14570251-14570887	enhancer	MCF7	GAT CTT GTC ATT GAC TCA CTC ATT CAT TCA ATC AGC AAA TAT TTA TTG AGT GAC TGC AAC GTG CCA GGA GCT GGA CAG TCA GCA AGA CAG ATG AAG TCT GTG CAT TCC ATG GTA TAG GGT	AAC CCA TAT TTG TTT ATC CAC CTT AAT TTA AGT TTG TTG TTA GTC ATA GCT GAA AAT GGG CTG GGT GTG GTG GCT CAC ACC TGT AAT TCC GGC ATT TTG GGA GGC CCA GGC AAG TGG ATC
chr5:34586108-34586774	enhancer	MCF7 & 786O	GAT CCA GGT GGC TTT CAA AAA TCT TAG TAC ATG AGA AAT ACC CCA TGG CCC ACT CTC TTA TTT CCC CTT TCA CCA TGG AAA CTC GCC AAG CTG AGT TTC TTG ACT GGG CTA CCT GCA CGA	CCA ACA CAC ACA CCT GCA TAC ACA TGC ACA TGC AAA TTG TTT GAA AGA AAC ATC ATA TGG CCA GGT ACG GCG GCT CAC GCC TGC AAT CTC AGG ACT TTG GAT GGC TGA GGC GGA AGG ATC
chr5:3926200-3927567	enhancer	MCF7	GAT CTG GGC TGG TCT CAG GTC GCA TTT CTT GGA TTT CCC CTC CAG GCG CTC AGC CCT TTT CTG CGC TAC GTG CCC TCC TGA TGC ATC CCT TCT CCC ATT CCA TCA GTG ACA CCC TTT CAC	TCC CAC CCG TCA ATA CAC ATT TGT TTC CCT CCC TGT CTC TGA GCT GCC CTC TTG ACT CTG GCC TAG CTT CTC CAA CTC TAA CCA CTT TTG TGT CTT TAT ACA ACA TGG CCT CCT CTG ATC
chr5:90575785-90576518	enhancer	MCF7 & 786O	GAT CCG CCC GCC TCG GCC TCC CAG AGT GAT GGG ATT ACA GGC GTG AGC CAC CGC GCC CGG CCT GTC TCA GGA ACT TTC AAA GGG GAG TGG CAG ACA GAA AAT GAG CAC GGT ATC TAA TCA	CTG ACG GGG GAA ATC CAC TGG CAG CGA GTC GGA TGA GTC AGC GCT GAG TCA CGC TCG GCG CTA CGT GCC GGC CTG GGC GGC CGC AGC CCG GGG CCC GCA GCC GCG GAG TCA GCA GAG ATC
chr6:106244048-106244211	enhancer	MCF7	GAT CAA CTT GAT TCC TTG TGT TTT ACG TGC TCT ACG TGA GAA AGG CTG AGA ACC CCA GAA GGT GAC ACT GGC TAC TCT GTA GGC ATG AAG TGA CTC CGT GAG TCA TTG CCA GAA TGT CAA	GCT GAG AAC CCC AGA AGG TGA CAC TGG CTA CTC TGT AGG CAT GAA GTG ACT CCG TGA GTC ATT GCC AGA ATG TCA AAG GGA TTT CTT AGT CAG GAA ACA GTC TCT GCC TTG GTG TTG ATC
chr6:164406604-164407478	enhancer	MCF7	GAT CAT CAA ATT GTA ATT TAT CAA AAA TGT ATT TTT ATC CAA GGG CTC TGG TGT CGT TAA GAA GTA TCT GCG TTT GTT CCA TCC AGG GTT AAC TGT CTT CTT GGA GGA GGG AGA AAG TCA	AGG CCT GGC TGC CCC CAA GTT GAT GGG GCC AGC TCC CTG CTG TCT TCC TGC CTG GCC CAC TAC CTG GGG CCT AGG CTT GCT CCT GCT CTT GCT AGT CTT GTG TGG ACC TTT CAG CTG ATC

chr7:101343709-101343829	enhancer	MCF7	GAT CTG GCT CTA ATG GGG TGG GGG TGG GGG GCC TGG GGA GAA GGG ACT GGT GTG ACC TGG GGC TGC AAA AAG GAT TCA ATG TCA TCC GGT GGG GCG GTG TGG AAT CAA AGG GGT CAG AGG	
chr7:140677611-140678311	enhancer	MCF7	GAT CTA ATT ACA TTG TTT TTC TTC AAG GGG CTA GTT GGT TCT GCA TCT CCA GTA TTG TGT GTG CGT GCA TAT GTG TGT GTA CCT GTC TCA AAT AAA GTA ACA ATT CCA GTT TGG CAA CCC	CTC CTG GGT TCA AGT GAT TCT CGT GCG TGT CAC CAC ACC CGG CTA ATT TTT GTA TTT TTA GTA GAG ACA GGG TTT CAC CAT GTT GGC CAG CTG GTC TCA AAC TCC TGA ACT CAA GTG ATC
chr7:1451023-1452826	enhancer	MCF7	GAT CTG TGT AAA CTG CAA TTG ATG CTG TGC CGC TTG ATA TTA ATA TCT TTT GTT TGT GTG TCA GGG TTC TGC AGA ATC TAA TTA AGA AGT GTT CTC TGC GTA TCC GGG GTG TTC GAA CAA	GTG CCC CGC CTC TTC AGA CTC CGC CCG ATT CCA GGC CAC GCC CCT TGT AGG CGT GAC CGA GGA CGC TGG TAC CCG GAC CTG GAC TTC GCA GGG TCT TGG AGG GGC GGG CAG TGG GGG ATC
chr8:128882979-128883308	enhancer	MCF7 & 786O	GAT CTT GGG CAG ATG GCT TAA CAT TTC TGG GCC TCA GTG ACC TTA CTT GCA AAG TTG TCT GGG TCA AGG CAA TGA TTA AAT GCG ATG ATG CAT GTA AAG CCC CAG CAT GCA GCC TGA AAA	GTC ACG TCT CCT TTC TGC CTC CTC CTC CTC CTC CTC TCT GTC CAG TGA CTA GGG AGG GCA GGA AGT TCA GAG GGT GAA ATG CTG AAT TAA TTC CTG TCC TCC TGG TCT ATA TTC CAG ATC
chr8:134308800-134310950	promoter	N/A	GAT CCA CAC CCG TTC CCG ACC CAG TCT GCA AGC CAG ACA CAC CCA CGC CCC CCT CTC CAG CCT CCT TGC CAG CGC CAC TCA CCT CTA CCA ACC CCT CAT CTA CAC ACT CGC GCG CAA TCT	ATT ATT AGG GCC AGT TTT GGA GAA GGG TTT TCT GGA ACA AAA GAA ATA TGG AGA GGG GCA AAC AGC ACC TCT AGG ACC CAG GAC ATC TGT GTT TCA GGC CTG TCT TTG ACA CTA TCG ATC
chr8:68737619-68737739	enhancer	786O	GTC CCT GAC TTA CCA CAA CAT TAC TTG AGG ACA GAA CTA CGC TCA TTT CCT TAA CAC AAA TAC ATC TCT TAC ACT ACA ACT TCT CAA AGT AGC AAA AAT GAA CCT TCT CTT TAA CAG ATC	
chr8:82105545-82107367	enhancer	MCF7 & 786O	GAT CTC TTT TCC TCA CAA AAC ATT ATT GAA AGT GAC TTT CTT CAG TTA CTT GGG ATT TTA TCA TCA CGA CCA TGT TAA CCA AAG AGG TAT CAT CTG GAC ACT GAA CCA GAA AGA AAA CCA	GTA TGG AAA TGC CTC ATC ATG GCT CCA TTT GGC TAT AAT AGC CTG ATT TGG CAC AAG CTT CTA ATA AAG CCT GAA GGT TAA TCA TAC TGC TGT GGA GCA GGA CTG GAG TTT TGC TAG ATC

chr9:121093990-121094567	enhancer	786O	GAT CAT GGG AGA AAC TCT GAA CTT GGG ACT AGT TGG GGG CAT AGA GGG AGT GTC AAT GCT TGG CTG GAA GTT GTC CCT CAG AGC AAT CAT TGC CCA GTG TTT TTG GCT TTA GCT CAA CCT	TTT TAG CTA AAA CAT TTT AAG CAC TAA TTA TAG GCC AAA CAT AGT GTT AAA CAT TTT GCA CAC ATC ATC TCA TTT AAT TTT CAC AAA ATT TTT GAA ATT AGT ACT ATT TTA ATT CAG ATC
chr10:71077459-71078709	promoter	N/A	GAT CTA CAA CTT CCT GGC TGT ATA AAG TCC AGC AAG ACT GAG GCC TCA CTG AGC TTC AAT TTC CTT ACC TGT CTA ACC AGA ATA GCA ACA ACT TGT TTG TAT ATT TGA GCT CAT TAC ATG	GGG GGA GGA GGA GGA GGA GGA GCC GCC GAG CAG CCG CCG GAG GAC CAC GGC TCG CCA GGG CTG CGG AGG ACC GAC CGT CCC CAC GCC TGC CGC CCC GCG ACC CCG ACC GCC AGC ATG ATC
chr10:74033444-74034056	promoter	N/A	GAT CCT TTC CAG AAA GGG GGC GTG GCG GTG GGT CGG GGT TCG ACT GCG AGC TTT CTG GGG CTC AAT GGA GGC GGG GCC CGG CCG CTG TCA CCG GGC AGG AGA GAA CGT TGC TTA CGT GCG	TGA GTT CAT CAG CAA ACG CCC TGG CGT CTG TCC TCA CCA TGC CTA GCC TTT GGG ACC GCT TCT CGT CGT CGT CCA CCT CCT CTT CGC CCT CGT CCT TGC CCC GAA CTC CCA CCC CAG ATC
chr10:99185459-99186091	promoter	N/A	GAT CAC ATT TTA CAG ATG AGG CTG AGA GGT TTC CCG ACC TGC CCA AAG ACA CAA AGC AAG TAG AAT GTA CGA GTG AAG ACT CGA ACA CAG GTC CGT GAA TCA ATC TCT GCG TGG GGA TGG	GAG CGG TGC GAG CGC GCA GGC GCG GCC GAC GGG GCG GGC TGC TAC TCC GGA ATC TGC TAA TCC CAG TCG GTG CCG CAT CCC CAG CCC GCC GCC ATG GCC GCC TAC AAA CTG GTG CTG ATC
chr11:18415792-18416140	promoter	N/A	GAT CCA AGC CTT CCT GAG AGT AAC CTC CCC AGG TTT CAT GGA TGA GGA AAC TGA AGG TCG TCC TGA CTC AGG CTC ATG GCT CCG ACC CCG GCT TCT GTG GTT GGA GGG CAG CAC CTT ACT	CAC ACG TGG GTT CCC GCA CGT CCG CCG GCC CCC CCC GCT GAC GTC AGC ATA GCT GTT CCA CTT AAG GCC CCT CCC GCG CCC AGC TCA GAG TGC TGC AGC CGC TGC CGC CGA TTC CGG ATC
chr11:69234614-69234815	enhancer	786O	GAT CAC TGG GCA GCC GTA CGT GTC CCA GGC AGC GGG AGC CCC GGC AGT GCC AGC TCC CAC ACC ATC CGG CTG TGA CAA CCT CAG GTC CTT GAT GAC AGG GAG CCT CTG CCA AGA CGG TCC	AGG TCC TTG ATG ACA GGG AGC CTC TGC CAA GAC GGT CCG AAA ATT GGG AAG GGG ACC CGC TCA TCT TTG TGG GGT CCC CAA GAG CTT CAG ATA AGT TTG CTG AGC ACC GGC TTC CTG ATC
chr11:71010452-71010944	enhancer	MCF7 & 786O	GAT CCA GAG CTC AGA GCC CAG CCC AAC ACT GGG GGA CGT GAC ACA GTG AGG AAG TAT GAA GCT CCA GCA GGA ACG CCA GCC AGA GGG AGC ACC AGG AAT GAG TCA TGT CAG CCT GGA CGT	TAC CTC CAG CGT CCT CAT GAG AGA AGA GAA GGG AAG GGC AGA GGA CAC GGA GGG GAT GTG ATG GAA ACA CAG AGC AGA CAC TGG GGT GAT GCA TCT ACA TTC CCA GGA GCA TCG TGG ATC

chr12:33096894-33097372	enhancer	MCF7	GAT CGT TCT GGT TTT AGC ACT GAA TGC TCA TGT ACT AGG AAA ACC TTC AGT TGC TGG CAA ACC AAG ATG GTT GGT TAC ACA GAT ATT TCT TTT AAT TAT TTA AAT CCT GTG TTG ATT AGA	GAT AAG CCA GAA AAG GTG AGT CAG TAC AGA ATG GCG TAA TAC AAG AAA TGT CCC CAA GGG CCA GAG TAG ACA GTG AGG CCA TCA CGT AGA CAT CAC TGT TAT TCA AGT CTG CTG ATG ATC
chr12:46494485-46496603	enhancer	786O	GAT CAG TTC CAT GAC AAT GTC CTT TAA CTA CAT CCT CAT TCA GCA GGA AGT AGG CAG AAT GAA CAC ATC GCC CAT GTT CCA CAG AAA TGG AAT GGA ATT TGG CAG TGG GGA GTT GTA ATG	GAC TTT GCA AGT GGT GAG CGA CTG AAC CTG CAT GGA GTT GCA ATA TTA GTG TTT TTT TTT TTT TTT TTT TTG AGA CAG AGT GTC GCT TTG TCG CCT AGG CTG GAG TGC AGT GAC GCG ATC
chr12:46887231-46888061	enhancer	MCF7	GAT CCC ACA TCT TTT TTT TGC TGA GTG TTT TCA TTT GAG GTT GAT TGT AAC TAT TTA AGG ACA TAT GGT AAC TAC TTA AGG GCT TAA TTA TTC AAT CAT GGA GAG TTG GGT GGA ACA TGG	ATT GTT ACA TAT TCA TAC ACA AGT ACT ATG AAG CAT AAC AGG AAA AAA CAG GTC TAC ATT TTC ATA AAA TTA AAT TCA CAC ATG CTC TGA TAG GCA AAA AGT TTT GTA CTT ACA TAG ATC
chr12:6643414-6644293	promoter	N/A	GAT CAC CTC CCA TCG GGC CAA TCT CAG TCC CTT CCC CCC TAC GTC GGG GCC CAC ACG CTC GGT GCG TGC CCA GTT GAA CCA GGC GGC TGC GGA AAA AAA AAA GCG GGG AGA AAG TAG GGC	GGC GGA GCC CCG CAC CCA GGC TGT GGC GCC CTG TGC AGC TCC GCC CTT GCG GCG CCA TCT GCC CGG AGC CTC CTT CCC CTA GTC CCC AGA AAC AGG AGG TCC CTA CTC CCG CCC GAG ATC
chr12:7023181-7024310	promoter	N/A	GAT CTG CTC TGT GCC GGT CCT CTG GGC AGT GTG GGG TGC AGA ATG GGG TGC CTA GGC CTG AGC GTT GCC TGG AGC CTA GGC CGG GGG CCG CCC TCG GGC AGG CGT GGG TGA GAG CCA AGA	CCT CAC CTC CCC CAG ACT TCC CTT GGA CTC CCC TCT CTC CTG CTC TTC CCC ACG GCG CCC CTC TCC GTT CGC GCT TCC TCC CCT CTG CTG CAC GGG GGA GAG ATG GAA GAA GTG GGG ATC
chr14:54404062-54405378	enhancer	MCF7	GAT CCA CCC GCC TCG GCC TCC CAA AGT GCT GGG ATT ACA GGC GTG AGC CAC CGC GCC CAG CCA GAA TGT TTC TTT ACG TTT TAG GTC TGC TGC TAG ATG TGT TTA GCC TAA CGA AAA TGC	CCA GTA CAA ACT TTT AGG CTA GGC TGG GTG GTG CAT CGG ACG CTG TGG ATG CTA TAA TTA CAT AAC AGT TCT CCT TTG ACC AGT GCT CAT CCA CGT ACG TCA CGT ACA CAC CAA TGG ATC
chr14:34420142-34420423	promoter	N/A	GAT CTG CCT TCG GGA ACC AGC GGG AGT GGT GCG GAG CTC CAC GAC CCG TTT CCG GAC TGG CCC GGC GAG CAG TGC GGC GCA AGG AGT CGG AGG GCG CCT TTT GGG GAT GGG AAG CCA CCA	GCC CTT AAG CCG GCC GAC TCC GCG CGT CAC GGG CTC CGC CCC GGG ACC GCC TCC GGG ACC GCC CTT TCC CCC GCC CCG CTG CCA GTC ACC CCC GCC TGC TCT GCC CCT CCG CGA AGG ATC

chr15:72523499-72525007	promoter	N/A	GAT CCG GAG CCA CGG CGC GTG CAG CTG CTG TGC AAG GAG CCA CTG CCT CAG CCT CAA GGT TAT CCC GCT GCG GGC CGC CGC AAT CCC TCC GCC GCC CCG CCC TCT GCC CAA TCC CGG CTC	CTA GGG AGG GTT AGA TTC CTC TCT ATC AGA GAA AGA GGG ATG GCA TAA AAG TCC CTC CCT GGC CCC TGA GCT GAG GAC AAA CTA AGA ACA GTG GTG GAC AAC ACT GGG ATG GCA AGG ATC
chr16:14458164-14459418	enhancer	MCF7 & 786O	GAT CTA TTA TGA TTT CAA AAT TTA AAG TTT TAA AAA ATG ACA TGC ATG GTA TGA TGT CGG CAA TTC GCT TTG AAA TAC TCC AGC CAA GAG AGG GAA GAG GCC GGA GAG GGA GCC TGC AAA	AAG TAT GTT GAG GCC AGG TGC AGT GAC GCA TGC CTG TCA TCC CAA CAT TTT GGG CAG CTG AGG CGG GAG CAT GGC TTG AGG CGA GGA GTT TGA GAA AAG CCT GGG CAA CAT CAT GAG ATC
chr16:30076906-30077297	promoter	N/A	GAT CGT GTT CTC GGC GCC CGC CCC TTC CTA GCG CGG CCT CTG GGC TGC GCC TCT CGG GGG CGG CCC GTA GCC CAG TCC GTC GCC TGC CAT TGG ACG CCG CCC GCT CCT CGT AAA GGA AAA	CCT TGA ATC CAC TCG CCA GCC CGC CGC CCT CTG CCG CCG CAC CCT GCA CAC CCG CCC CTC TCC TGT GCC AGG TGA GCG CCC CTC TTC ACG TGC GGG GAC CAG GGA CCG TGG AGA GGG ATC
chr17:26903868-26904491	promoter	N/A	GAT CCG CAA ACA GAT GAG GCT GCA GCC CTG GCT CCC TCT GGT AAA TGA GGC TGC GGA TGT CGC AGA GCT ACG TGA CTC CTC CGG GGG CGT GGC CTG CAT GCC CCG CCC TGA CCA CAC CCC	TCC TCC AGG CGG TGA GCC AGG GAA CAA GCA GTC CAG TGC CCA GAG TTT TGG TGA AAG CAG AGG AAG GGA GGA AGG GCA GGC AGC CTT GGC TTT GGA TGC AGC AGG AGG TGG AGA GGG ATC
chr19:34855951-34856857	promoter	N/A	GAT CAG CGG CCG CGG GCA AGG TCG CTC AGC GGG CAC CCG GCC TGG GTA TCG GGG CGC GGG TCG GGG GCG GGG CCG GGG CTC AGG GGT GGG GCC GGG CCG GGC CGG GCC GGG CGC CTG CGC	GGG TGG AAG GAT GGA GAC GTC GGG CGT GAT TCC TGC AGA AGA CAC CTG TGC ACG ACC GGG AAG TGG GAA GGA GTG TTT GAG CAA ATC ATG GGC GGG AGT GCG GCC AAG GGA GCT CTG ATC
chr20:40292823-40293501	enhancer	MCF7	GAT CTT GCC ATT GCA CTC TAG CCT AGG CGA CAG AGT GAG ACT CCA TCT CAA AAC AAA CAA AAA ATG AAA ACC ATG AAT TAG AGC TAT ACC AAT GAA CAT GGA TAA TAT TTC ACA ATG TAT	CTC TTA AAG ATT TCT GGA TGC GCA ACA AAA TGC TCC CAT TGG TCA GAA CTT AGT CAC ATG AAC ACA TCT AGC TGC AAA GGA GAA TGG GAA ATG TAG TCT TTA TTC TGT GTG GCT ATG ATC
chr21:45719837-45721899	promoter	N/A	GAT CCC TGC TCC GTC CAG GGT CCG TCC TGC GAG GAG GCG CCG GGG GCG GGC CCG GGG CGG GGC GGG CGG AAG GCG CGC GCG GGG CGG GGC GGG GAC GGC GAC GCG GCG CAG GCG GCG GGA	ACT AAA CAG AGA AGG GGG GCT CGC TGG AGG TTG CTG AAG GGC CAG CCT GGC AGG TGG TTC TTG GGC CAG CGG GGA GGG GCA CCC AGG GTG ACA GGC GGT TCT GGA TGC TGG TGG AGG ATC

chrX:77359452-77360120	promoter	N/A	GAT CTT CGC CGC TAC CCT TGT GGG CCC CCC GGC GAC GCT TCC TGC TCC GCC CCT AAG TCG GGA AGG TTC CTT GCG GTT CGC GGC GTG CCG GAC GTG ACA AAC GGA AGC CGC ACG TCT CAC	TTT TGG CTG GGC CCC AGG GGT CCT AGG CTT GGA GGG CGA GGC TGC TCA CGG GTT TGG TGG TTT CTA GCC GCA TTT TCC CCA GCC CAG AAA GCA CCC GAA GTC ACC CTT CGG GGA TGG ATC

Table S2: Number of unique informative reads per bait site

Bait Dpn2 fragment	786O 1	786O 2	MCF7 N 1	MCF7 N 2	MCF7 H 1	MCF7 H 2	786O+ VHL N	786O+ VHL H
chr1:115666026-115666146	5243	3607	3689	1571	3732	854	16005	11645
chr1:43424102-43424222	12964	3920	4019	2736	4212	1979	7552	6147
chr1:8086133-8086253	11357	4356	5337	2719	4763	1562	10437	8839
chr1:8938715-8938835	10815	4440	4762	3551	4169	2294	11164	8223
chr10:71077459-71077579	8505	3743	4963	3155	4664	1730	9063	6455
chr10:74033444-74033564	15189	4908	5149	3329	5276	2546	13723	12315
chr10:99185459-99185579	12626	5841	6386	3552	5831	2344	17974	13654
chr11:18415792-18415912	6492	3020	3953	2679	3646	1902	9218	7731
chr11:69234614-69234734	11220	5318	6635	3280	5788	1793	12119	10466
chr11:71010452-71010572	14073	7515	9161	5627	9107	3738	18737	14353
chr12:33096894-33097014	3000	2175	2843	1136	2914	647	10754	7958
chr12:46494485-46494605	3164	2862	3829	1446	3695	921	6560	5988
chr12:46887231-46887351	1163	1054	951	454	822	278	17134	13450
chr12:6643414-6643534	13545	5525	7835	4259	7108	2676	10645	8094
chr12:7023181-7023301	12402	4612	7556	3598	7990	2242	7738	5483
chr14:54404062-54404182	1641	1313	2618	1215	2701	668	7471	5171
chr15:72523499-72523619	8716	4282	6239	3641	6200	2560	8673	6166
chr16:14458164-14458284	6461	3500	2687	1247	2437	652	18226	15942
chr16:30076906-30077026	17734	6796	8412	6912	7403	4557	10951	9137
chr17:26903868-26903988	12900	5259	9389	5845	9076	4009	5979	4108

chr19:34855951-34856071	11413	3583	6604	4206	6842	2865	9165	7314
chr2:173420581-173420701	8303	2659	3258	2678	2671	1607	6512	6458
chr2:70823890-70824010	7962	5614	5584	3118	5192	2048	15114	12420
chr2:75060942-75061062	13308	4471	5955	4578	5013	2950	7688	6142
chr20:40292823-40292943	2944	2653	2890	1288	2849	851	14388	10224
chr21:45719837-45719957	22265	5495	8381	5560	8190	3958	7937	6348
chr3:150063120-150063240	2233	2286	2745	1245	2514	732	19912	15680
chr5:14570251-14570371	4955	6846	11660	4136	11622	2448	17207	11954
chr5:34586108-34586228	7048	4418	3796	1741	3507	1034	18809	13791
chr5:3926200-3926320	4219	1876	3155	1052	3514	662	5325	3300
chr5:90575785-90575905	5380	2849	4683	2930	4869	2112	7683	6123
chr6:106244048-106244168	4153	2681	3602	1453	3573	778	9461	6243
chr6:164406604-164406724	2835	1489	1185	425	1347	267	11145	8316
chr14:34420142-34420262	6016	2064	3566	2387	3682	1428	7347	5854
chr7:101343709-101343829	2858	1452	1833	769	1720	444	926	547
chr7:140677611-140677731	2511	4153	5194	1749	5510	962	15462	11427
chr7:1451023-1451143	4727	1971	5125	2343	4722	1384	3379	2220
chr8:128882979-128883099	9505	6450	4212	1631	4150	941	23985	19157
chr8:134308800-134308920	15716	7815	6016	3129	5588	1819	18875	14552
chr8:68737619-68737739	1044	1400	432	147	494	95	7348	6374
chr8:82105545-82105665	3782	3727	1966	735	2279	480	22905	19110
chr9:121093990-121094110	5386	3595	2164	899	2273	567	14014	12098
chrX:77359452-77359572	5561	2413	2114	1493	2273	1013	8057	5936

References

1. Jin F, Li Y, Dixon JR, Selvaraj S, Ye Z, Lee AY *et al* A high-resolution map of the three-dimensional chromatin interactome in human cells. *Nature* 2013*;* **503:** 290-294.
