# Supplementary material for: Capture‐C reveals preformed chromatin interactions between HIF‐binding sites and distant promoters
Source: EMBO Rep. 2016 Aug 9;17(10):1410–21. doi: 10.15252/embr.201642198 (PMC5048371; doi:10.15252/embr.201642198)
Supplement: Supplementary file 2 — Expanded View Figures PDF [file EMBR-17-1410-s002.pdf]

Expanded View Figures

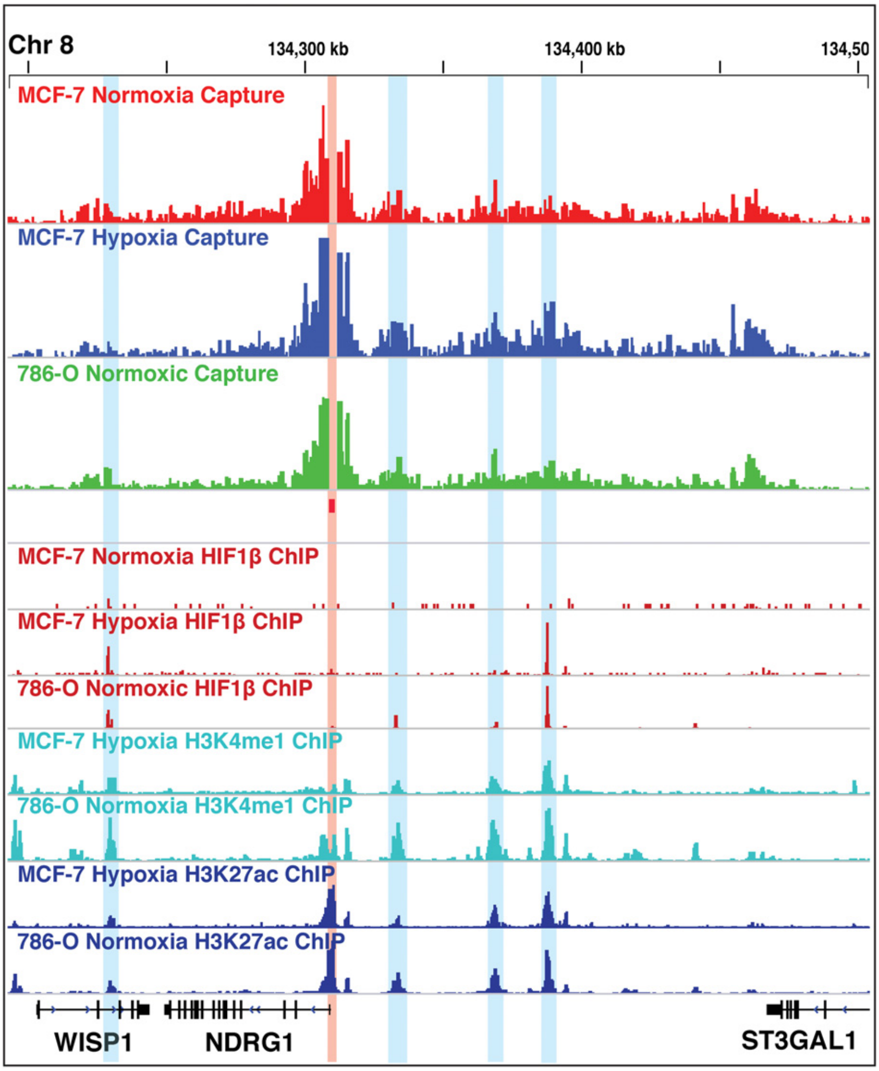

Figure EV1. Promoter of hypoxia-regulated gene NDRG1 interacts with multiple HIF-binding sites.
Capture-C and ChIP-seq tracks at the NDRG1 locus, showing that the NDRG1 promoter (bait site highlighted by pink shading) is in close contact with multiple strong HIF-binding sites (blue shading).

© 2016 The Authors

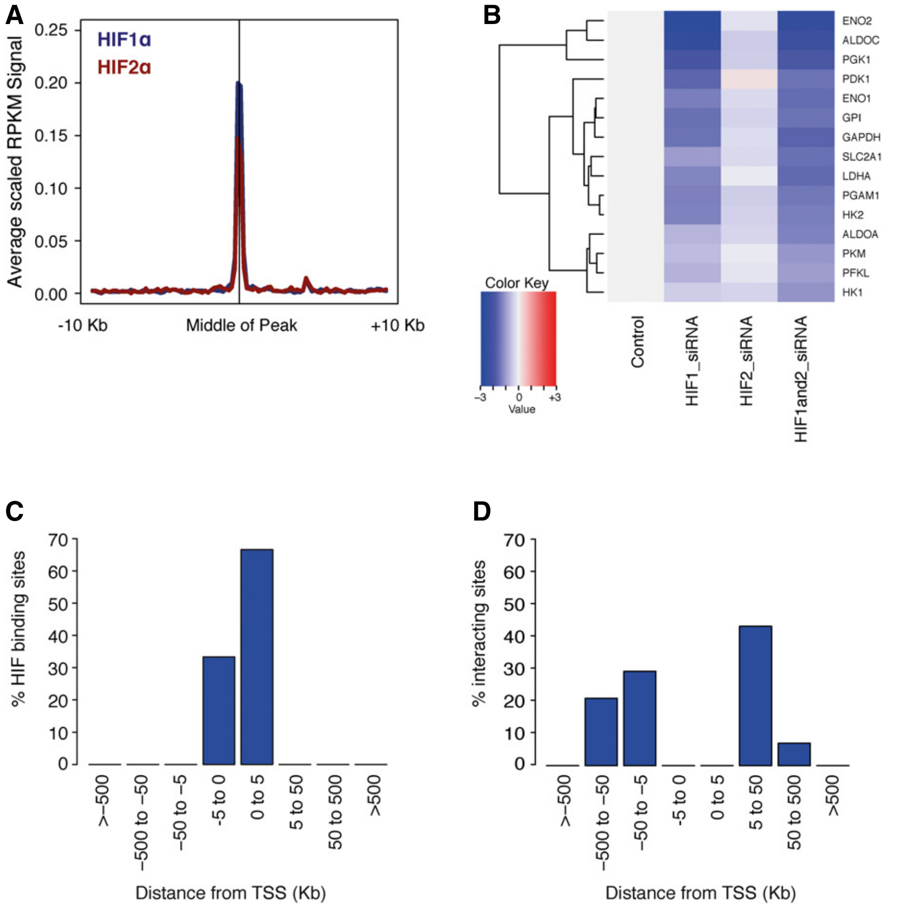

Figure EV2. HIF regulation of the glycolysis pathway.

A Averaged HIF1α (blue) and HIF2α (red) ChIP-seq signals (RPKM) at the HIF-binding sites closest to the HIF-regulated glycolysis pathway genes highlighted in Appendix Fig S5.

B Heatmap showing the changes in expression of HIF-regulated glycolysis pathway genes (normalized to the control siRNA) when either HIF1α or HIF2α or both are knocked down by siRNA. Genes were ordered by row according to hierarchical clustering.

C Frequency histogram showing the distribution of genomic distances between the promoters of glycolytic genes and the closest HIF-binding sites.

D Frequency histogram showing the distribution of genomic distances between the promoters of glycolytic genes and all enhancers looping to them.

 © 2016 The Authors

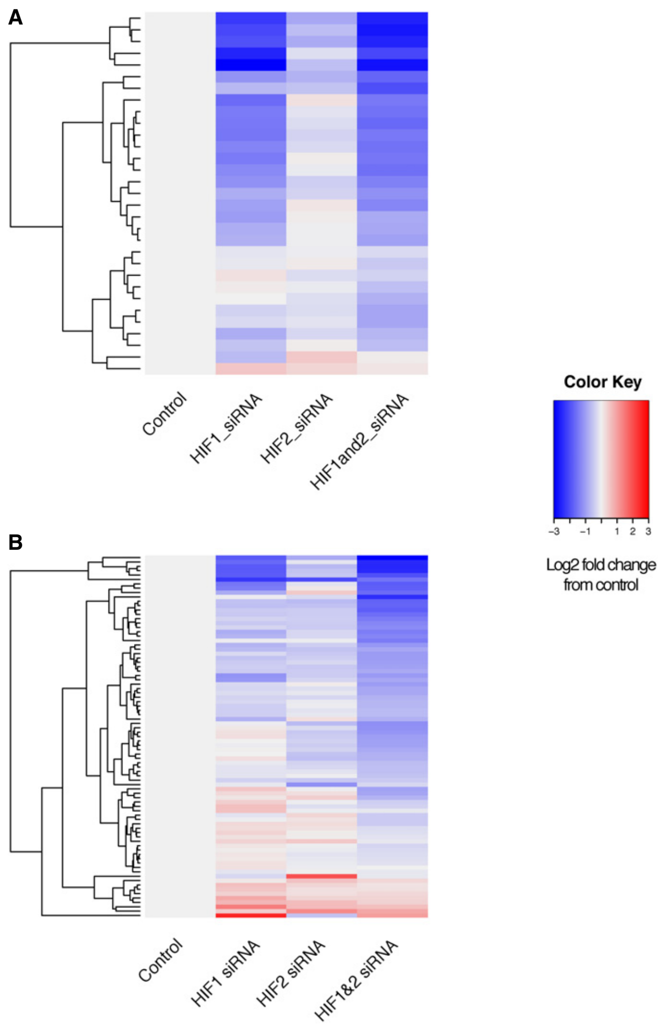

Figure EV3. Differential transcriptional activation activity of HIF1 and HIF2.

Binding sites bound both by HIF1 and HIF2 were identified and classified by the distance from the binding site to the closest TSS. Expression of the closest gene (normalized to the control siRNA) is shown for MCF-7 cells treated with control siRNA, HIF1α siRNA, HIF2α siRNA or HIF1α and HIF2α siRNA combined. Genes were ordered by row according to hierarchical clustering.

A Genes bound by HIF at their promoters (2 kb or less).
B Genes bound by HIF at distal enhancers (10 kb or more).

© 2016 The Authors

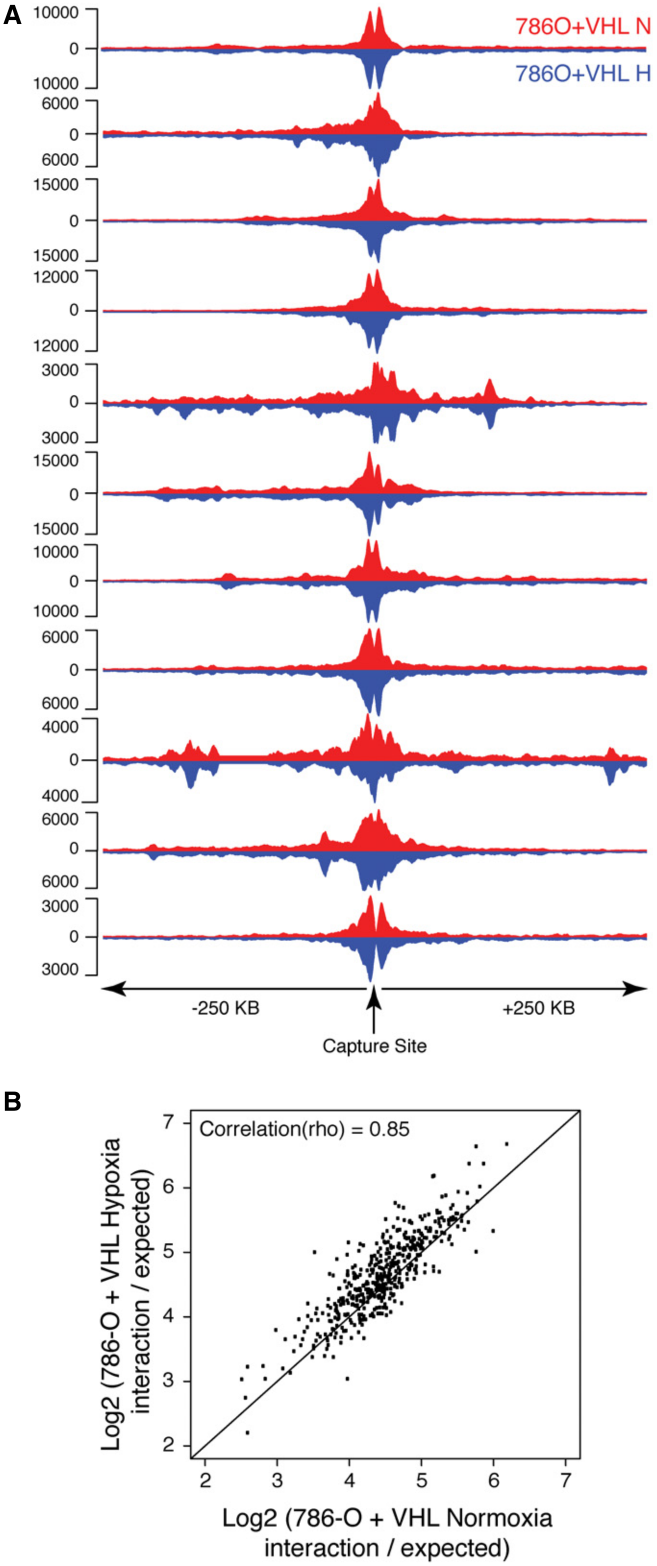

Figure EV4. Chromatin looping is pre-established in normoxia in 786-O cells reconstituted with wtVHL.

A Capture-C tracks from normoxic (red) and hypoxic (inverted in blue) 786-O cells reconstituted with wtVHL are shown for each HIF-binding site captured.

B For each site that looped to a HIF-binding site, the normalized interaction frequency in hypoxia (vertical axis) was plotted against that in normoxia (horizontal axis).

 © 2016 The Authors

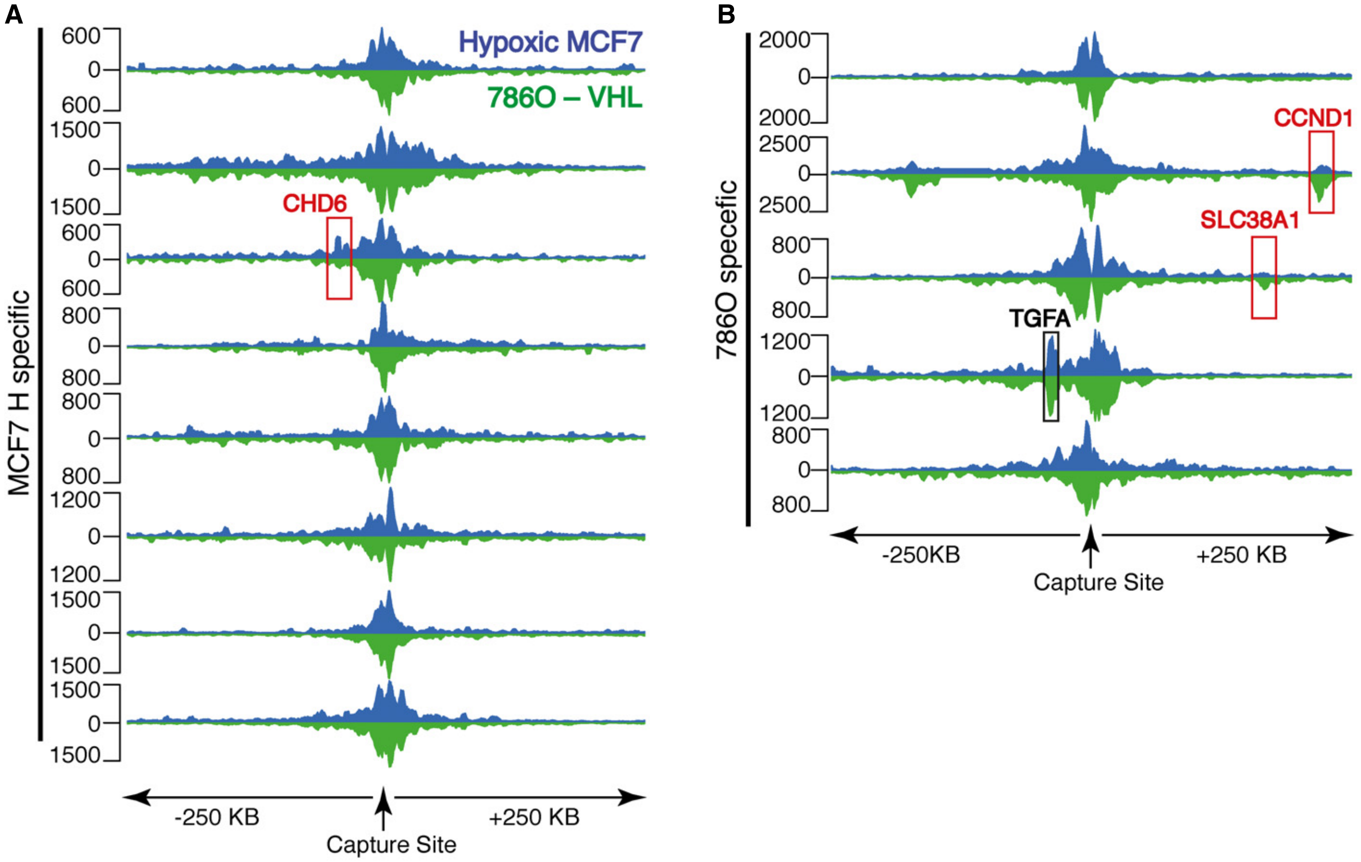

Figure EV5. Patterns of chromatin interaction from cell-specific HIF-binding sites.

A, B Capture-C tracks (averaged across the two replicates) from hypoxic MCF-7 cells (blue) and VHL-defective 786-O cells (inverted in green) are shown for each HIF-binding site that was bound exclusively in one cell line or the other. Three promoters showing significantly different interaction with the HIF-binding site are highlighted in red, otherwise association is largely conserved between the two cell types. One promoter showing similar interaction frequency despite differential HIF binding is highlighted in black.

© 2016 The Authors
