# Supplementary material for: Capture‐C reveals preformed chromatin interactions between HIF‐binding sites and distant promoters
Source: EMBO Rep. 2016 Aug 9;17(10):1410–21. doi: 10.15252/embr.201642198 (PMC5048371; doi:10.15252/embr.201642198)
Supplement: Supplementary file 3 — Table EV1 [file EMBR-17-1410-s003.docx]

## Table EV1: Effect of HIF siRNA on promoter bound gene expression

| **gene id** | **Control** | **HIF1 siRNA** | **HIF2 siRNA** | **HIF1and2 siRNA** |
| --- | --- | --- | --- | --- |
| ANKRD37 | 622.0614728 | 106.6734666 | 534.9113947 | 146.5600089 |
| BHLHE40 | 23317.94678 | 19316.49841 | 20930.36082 | 12180.85574 |
| C3orf14 | 6507.974099 | 5908.281034 | 6260.585681 | 5420.624739 |
| C8orf58 | 1334.404434 | 490.1090443 | 1379.094824 | 468.4838265 |
| CITED2 | 10703.86131 | 6690.56004 | 7284.658974 | 2683.358789 |
| CRKL | 11134.34987 | 7653.875414 | 11559.56903 | 7160.961851 |
| ENO1 | 130159.482 | 51119.31823 | 107892.0716 | 44502.80156 |
| FAM129B | 14736.80144 | 11298.65914 | 12309.67617 | 7756.832836 |
| FAM162A | 24137.08614 | 3028.767905 | 15857.3255 | 3517.581238 |
| FEM1C | 2959.471219 | 1703.277037 | 2930.785071 | 1494.160098 |
| GAPDH | 594745.8177 | 211899.6174 | 498283.8184 | 185284.088 |
| GBE1 | 15054.98864 | 3567.701869 | 9748.410564 | 2299.764611 |
| GLTSCR2 | 7496.666493 | 6969.55615 | 7999.283958 | 5288.784019 |
| GPI | 84013.53063 | 29552.28044 | 65345.39687 | 29993.97017 |
| GTF2IRD2B | 15.4139126 | 9.786773408 | 21.72836571 | 15.90448393 |
| HILPDA | 13220.73304 | 3390.118818 | 7313.636304 | 2272.988399 |
| KLF10 | 10150.06145 | 11673.40831 | 8588.385065 | 7724.965198 |
| LDHA | 310306.2825 | 128668.5079 | 291094.1815 | 103441.1859 |
| MAB21L3 | 3686.127099 | 3887.748011 | 3497.977114 | 2407.498066 |
| MTFP1 | 851.0681743 | 412.0301658 | 884.1348068 | 460.6020969 |
| P4HA1 | 30290.54025 | 13298.73321 | 22817.65469 | 11636.15567 |
| PDK1 | 4853.180482 | 1544.299218 | 5580.61139 | 1734.874962 |
| PPP1R3C | 1725.257218 | 357.080724 | 921.8590179 | 287.3762269 |
| RSBN1 | 6950.573589 | 3902.803862 | 6773.48458 | 3686.651248 |
| SCD | 969030.8475 | 436461.8869 | 565113.3694 | 295316.1346 |
| STC2 | 49399.3879 | 28027.40423 | 40389.42344 | 30128.14607 |
| TFRC | 8849.787821 | 8797.65371 | 7654.18728 | 5104.114737 |
| TRIM33 | 23587.69025 | 33874.5362 | 29906.87708 | 26081.15578 |
| TRIOBP | 7371.153205 | 2674.956855 | 6553.241297 | 2479.404967 |
| UBC | 94633.71641 | 53425.23148 | 74504.08816 | 41560.44404 |
| WSB1 | 7852.287476 | 3945.376609 | 8567.992842 | 3131.626747 |
