# Supplementary material for: Capture‐C reveals preformed chromatin interactions between HIF‐binding sites and distant promoters
Source: EMBO Rep. 2016 Aug 9;17(10):1410–21. doi: 10.15252/embr.201642198 (PMC5048371; doi:10.15252/embr.201642198)
Supplement: Supplementary file 4 — Table EV2 [file EMBR-17-1410-s004.docx]

## Table EV2: Effect of HIF siRNA on enhancer bound gene expression

| **gene id** | **Control** | **HIF1 siRNA** | **HIF2 siRNA** | **HIF1and2 siRNA** |
| --- | --- | --- | --- | --- |
| AHNAK | 68786.78697 | 45077.59306 | 46154.79199 | 20976.48691 |
| ARRDC3 | 6311.99721 | 7580.994519 | 7479.833947 | 4610.788518 |
| ATP9A | 16518.20934 | 16531.13309 | 12739.72798 | 8359.650467 |
| BCCIP | 9204.30781 | 8160.519193 | 7489.747139 | 6110.315436 |
| BLCAP | 1619.561817 | 2635.324697 | 2485.809148 | 2099.385298 |
| BZW1 | 43647.79651 | 39484.61441 | 42666.34249 | 29845.65407 |
| C1D | 1004.106307 | 1136.923734 | 946.1646833 | 876.835217 |
| C1orf116 | 767.3926488 | 625.581737 | 796.9726288 | 370.8007138 |
| CAV3 | 1.100993757 | 0.889032477 | 4.300598561 | 1.017586258 |
| CCNL1 | 2510.265766 | 2717.256592 | 2306.263387 | 2145.662849 |
| CLIC6 | 24.22186266 | 21.71524382 | 30.56660685 | 17.40448393 |
| CUX1 | 14355.8576 | 10893.71904 | 12763.19372 | 9061.14805 |
| CYP1B1 | 43161.15727 | 48947.2228 | 41509.08227 | 43146.02844 |
| DAAM1 | 11750.90637 | 9276.131598 | 10119.53378 | 6259.659651 |
| DAZL | 1.100993757 | 1.631128901 | 1.054423378 | 1.017586258 |
| DDIT4 | 53270.48195 | 24183.76152 | 39562.18129 | 25343.38459 |
| DEGS2 | 658.3942668 | 233.8688418 | 587.2566733 | 184.6503569 |
| DIRC3 | 63.85763792 | 29.87830449 | 51.16931525 | 34.41448542 |
| DNAJB6 | 16159.28537 | 11619.86546 | 12005.60381 | 8882.634963 |
| DNM2 | 15108.93733 | 14618.07531 | 11061.23608 | 7786.335587 |
| DPT | 4.403975029 | 0.889032477 | 1.054423378 | 1.517586258 |
| DSP | 148873.0729 | 100606.6491 | 95049.49352 | 43768.07781 |
| EGFR | 2378.146516 | 1738.271934 | 1768.920782 | 674.2576218 |
| ELF3 | 9390.375755 | 5966.305702 | 6386.282206 | 4972.060231 |
| ELMSAN1 | 7547.312206 | 7629.569529 | 5892.410819 | 4097.046066 |
| FAM65C | 30.8278252 | 176.9855956 | 21.24260654 | 61.94965793 |
| FBXW7 | 1735.166161 | 3193.660488 | 2166.15869 | 2173.730108 |
| GADD45B | 2635.779055 | 2024.968901 | 1858.163636 | 1915.112507 |
| GJA1 | 309.3792458 | 114.7445188 | 336.7160201 | 89.97966238 |
| GPR135 | 23.1208689 | 21.62323539 | 10.12970855 | 18.67586258 |
| GPR37L1 | 772.8976176 | 463.1606575 | 579.9340653 | 359.3034651 |
| GSE1 | 39440.89936 | 37454.76562 | 31018.20393 | 25715.01461 |
| HERC1 | 2618.163155 | 3122.778461 | 3018.408859 | 2189.229104 |
| HEY1 | 1200.083195 | 859.4733074 | 878.4248224 | 389.8358863 |
| IGFBP3 | 6026.839827 | 1666.773405 | 3303.864213 | 432.0820939 |
| INHBA | 159.6440948 | 231.0756696 | 156.7669993 | 82.48724715 |
| IRX1 | 1.100993757 | 1.407016741 | 1.540182544 | 1.017586258 |
| ITGA5 | 8268.463117 | 4957.125899 | 6699.711966 | 3335.276769 |
| KCNMA1 | 339.1060772 | 364.8046038 | 286.8031589 | 150.2082837 |
| KIAA0195 | 2785.514206 | 2003.110696 | 2280.972924 | 1386.543835 |
| KLHDC4 | 1328.899465 | 1471.467168 | 1304.965239 | 1066.912812 |
| KRT80 | 39375.94073 | 59585.42928 | 33923.73112 | 30906.17132 |
| LEKR1 | 63.85763792 | 57.76926419 | 65.63584292 | 58.85414187 |
| MAF | 194.875895 | 209.381287 | 289.6440656 | 241.6455235 |
| MDGA2 | 122.2103071 | 133.4394638 | 161.4108891 | 72.590348 |
| MRPS18A | 1856.275475 | 2030.101473 | 1668.057136 | 1565.469735 |
| NANOS3 | 5.504968786 | 5.341611024 | 5.272116892 | 2.017586258 |
| NAP1L5 | 31.92881896 | 86.13371118 | 50.39982758 | 48.44965793 |
| NCOR2 | 38691.12262 | 26380.11692 | 29324.17335 | 18758.0448 |
| NDRG1 | 105313.3559 | 28961.33023 | 66021.94632 | 18696.57034 |
| NUP210 | 8774.920245 | 13315.56638 | 11087.05898 | 9853.308408 |
| P2RY2 | 1937.749013 | 2212.415234 | 1387.107637 | 883.2497024 |
| PFKFB3 | 27942.12056 | 7766.88914 | 19344.06253 | 7153.436011 |
| PKP2 | 2731.565512 | 3160.304857 | 2003.626571 | 1348.206276 |
| PNMA2 | 97.98844439 | 117.9845281 | 113.3802946 | 51.53758922 |
| PPP4C | 4321.400497 | 4946.4522 | 4487.830744 | 3879.541567 |
| PRDM1 | 122.2103071 | 71.69249555 | 141.6722446 | 70.91690212 |
| PSMD6 | 61579.68183 | 88468.78432 | 72171.74427 | 69754.0697 |
| PXDN | 20059.00526 | 15810.11948 | 15042.13507 | 7289.063465 |
| QSOX1 | 23228.76629 | 13189.35816 | 18972.1038 | 8654.434746 |
| R3HCC1 | 706.8379921 | 690.0136495 | 787.458269 | 514.7427869 |
| RNF19A | 6060.970633 | 5361.381899 | 5449.192002 | 3956.500557 |
| SCARB1 | 13194.30919 | 10539.18043 | 9572.596318 | 5581.996206 |
| SHANK2 | 1241.920958 | 1247.478255 | 848.9323596 | 709.4938279 |
| SLC28A1 | 17.61590012 | 20.44774697 | 11.51575211 | 10.28138013 |
| SLC2A3 | 145.331176 | 80.54272503 | 201.5268692 | 46.76621058 |
| SLC38A2 | 29185.14252 | 21825.49038 | 27923.02538 | 17683.9562 |
| SLC45A1 | 73.76658173 | 70.40275021 | 60.95573249 | 13.88689768 |
| SLC9A9 | 2.201987514 | 2.960969535 | 5.272116892 | 4.08793129 |
| SNAPC1 | 3609.057536 | 2716.011343 | 2574.792021 | 1865.550081 |
| SPTSSA | 7480.151587 | 7745.84882 | 7007.426512 | 6187.019965 |
| SUMF1 | 1606.349892 | 2140.721351 | 1585.469628 | 1197.146602 |
| TLE1 | 2597.244273 | 2125.297468 | 2459.287182 | 1594.439061 |
| TMEM189 | 7328.214448 | 6181.27208 | 6785.518686 | 3910.33477 |
| TPCN2 | 190.47192 | 244.0579553 | 196.6097147 | 163.4017326 |
| TRAM2 | 1964.172863 | 1397.950483 | 1449.205526 | 795.3452184 |
| TSC22D2 | 9455.334387 | 9055.722504 | 5992.197911 | 4119.348787 |
| UBE2E1 | 10268.96877 | 7697.864755 | 9461.811841 | 6559.902698 |
| WISP1 | 28.62583769 | 9.037260821 | 26.02896427 | 5.158276322 |
| ZFAT | 897.3099121 | 702.2508244 | 723.9988851 | 522.4689916 |
| ZFP36L1 | 16600.78387 | 21826.02256 | 19447.21572 | 20648.59782 |
| ZNF395 | 20863.8317 | 5688.525469 | 14345.47756 | 3782.258848 |
| ZNF572 | 312.682227 | 259.2234205 | 223.0403259 | 152.0851799 |
